# Applying Target Capture Sequencing to Unravel the *Anthurium* Section *Pachyneurium* (Araceae), with Emphasis on Brazilian Species

**DOI:** 10.3390/plants15060866

**Published:** 2026-03-11

**Authors:** Mel C. Camelo, Georgios J. Pappas, Micheline C. Silva, Lívia G. Temponi, Marcus A. N. Coelho, José F. A. Baumgratz, Mónica M. Carlsen

**Affiliations:** 1 Departamento de Botânica, Instituto de Ciências Biológicas, Universidade de Brasília (UnB), Brasília 70910-900, Brazil; silvamicheline@gmail.com; 2 Diretoria de Pesquisa Científica (DIPEQ), Instituto de Pesquisas Jardim Botânico do Rio de Janeiro, Rio de Janeiro 22460-030, Brazil; mnadruz@jbrj.gov.br (M.A.N.C.); jbaumgra@jbrj.gov.br (J.F.A.B.); 3 Departamento de Biologia Molecular, Instituto de Ciências Biológicas, Universidade de Brasília (UnB), Brasília 70910-900, Brazil; gpappas@unb.br; 4 Herbário UNOP, Universidade Estadual do Oeste do Paraná, Cascavel 85819-110, Brazil; liviatemponi@yahoo.com.br; 5 Plant Taxonomy and Evolution Department, Biodiversity Research Division, Missouri Botanical Garden, St. Louis, MO 63110, USA; monica.carlsen@mobot.org

**Keywords:** Alismatales, Angiosperms353, Hyb-Seq, neotropical region, PAFTOL, phylogenetics

## Abstract

*Anthurium* (Araceae) is one of the most species-rich Neotropical genera, yet its infrageneric classification remains unresolved. This study tests the monophyly of the morphologically defined *Anthurium* sect. *Pachyneurium* diagnosed by rosulate habit, involute prefoliation, and absence of a collective vein with a focus on Brazilian species. Using target capture sequencing (Angiosperms353 probe set), we generated a phylogenomic dataset for 35 *Anthurium* species (18 from sect. *Pachyneurium*) and conducted maximum likelihood and coalescent-based analyses. Our results demonstrate that sect. *Pachyneurium* is not monophyletic as traditionally circumscribed. Brazilian species previously assigned to the section are recovered in three geographically structured and strongly supported lineages: Amazonian, Atlantic Forest, and Caatinga/Cerrado. The Atlantic Forest lineage is unexpectedly resolved as sister to *A. coriaceum* (sect. *Urospadix*), revealing an evolutionary relationship not predicted by morphology. Divergence-time estimates place the origin of crown *Anthurium* in the Paleocene (~62 Ma), with diversification of the Brazilian lineages occurring during the Miocene (20–3 Ma), coinciding with major geoclimatic events in South America. Our findings indicate that key diagnostic morphological characters are homoplastic and provide a phylogenomic framework for revising the infrageneric classification of *Anthurium*. By identifying evolutionarily distinct lineages, this study also contributes to prioritizing conservation efforts in threatened Neotropical biomes.

## 1. Introduction

Araceae Juss. (Alismatales) comprises approximately 150 genera and over 4600 species [1,2], with *Anthurium* Schott standing out as the richness Neotropical genus (~1300 species) [3,4]. Despite its diversity, the genus remains taxonomically challenging. Its current infrageneric classification organizes species into 20 sections based primarily on morphological characters, yet phylogenetic assessments indicate that only a minority of these sections are monophyletic [5,6,7,8,9,10], revealing discordance between morphology-based taxonomy and evolutionary history.

This discordance is particularly evident in *Anthurium* sect. *Pachyneurium* (Schott) Engl., a morphologically distinct group recognized by rosulate habit, involute prefoliation, and eucamptodromous venation lacking a collective vein [11,12,13,14]. With 21 described taxa in Brazil, the section shows notable diversity across the Amazon, Atlantic Forest, Caatinga, and Cerrado domains [12,13,14]. However, previous phylogenetic studies using few plastid and nuclear markers yielded largely unresolved species-level relationships due to limited variability and phylogenetic signal [4,8,9], and the monophyly of sect. *Pachyneurium* remains untested with comprehensive sampling.

Resolving relationships in megadiverse groups like *Anthurium* requires phylogenomic data to overcome limitations of few markers [9]. Target capture sequencing (Hyb-Seq) enables enrichment of hundreds of conserved loci, even from herbarium material [15,16,17,18,19,20,21,22,23]. The Angiosperms353 probe set has been successfully applied across monocot lineages with complex radiations, including Asparagaceae [24,25], Orchidaceae [26,27], Arecaceae [28], and Poaceae [29], as well as in other Araceae genera [30,31], indicating its potential for resolving infrageneric relationships within *Anthurium*.

Here, we present the first phylogenomic analysis of *Anthurium* focusing on sect. *Pachyneurium*, with emphasis on Brazilian species. Our objectives were to (1) generate a genomic dataset using Hyb-Seq and Angiosperms353; (2) reconstruct the phylogeny of sect. *Pachyneurium* and test its monophyly; and (3) estimate divergence times for the genus and its main lineages. Based on previous morphological and molecular evidence, we hypothesize that sect. *Pachyneurium* is not monophyletic and that Brazilian species form geographically structured lineages reflecting their evolutionary history. By identifying evolutionarily coherent lineages, this study provides a phylogenetic framework to inform future infrageneric revisions of *Anthurium*.

## 2. Material and Methods

### 2.1. Taxon Sampling and Genomic DNA Extraction

A total of 36 Araceae species were sampled in this study, with 35 species from the genus *Anthurium* comprising 14 sections, and one outgroup species, *Pothos scandens* L. *Pothos scandens* was selected as the outgroup based on well-established phylogenetic relationships within Araceae, where *Pothos* (subfamily Pothoideae) is consistently resolved as the sister lineage to *Anthurium* [7,8].

Among the *Anthurium* species, 18 belong to sect. *Pachyneurium* (Schott) Engl. This section comprises approximately 120 species distributed across the Neotropics, of which 21 are recorded in Brazil [11,12,13,14]. Our sampling includes 11 species that occur in Brazil, representing 52% of the Brazilian species (including eight endemics) and 15% of the section’s total diversity. Species were selected to encompass the major geographic centers of diversity of the section and to represent the breadth of morphological variation within the group: Brazilian Amazon, Atlantic Forest, Caatinga/Cerrado, Caribbean, Central America, and the Andean region. To ensure accurate nomenclatural representation, the type species of sect. *Pachyneurium*, *A. crenatum* (L.) Kunth, was included in the sampling.

To test the monophyly of sect. *Pachyneurium* within a broader phylogenetic context, representatives of 13 additional sections of *Anthurium* were included as ingroup references (see Table S1). These sections are sect. *Schizoplacium* Schott (Engl.), sect. *Episeiostenium* Schott (Engl.), sect. *Leptanthurium* Schott (Engl.), and sect. *Multinervia* Croat (Carlsen & Croat), each represented by two species, while the remaining nine sections are each represented by one species.

A single accession per species was used, as the primary objective of this study is to resolve phylogenetic relationships at the sectional and interspecific levels, not to investigate population structure or infraspecific variation. While population-level sampling across the full distribution range of each species was beyond the scope of this study, each species is represented by material collected from its typical area of occurrence, covering the major biogeographic regions where the section occurs.

All samples were obtained from fresh leaf tissue collected during field expeditions in 2019–2020 across the main Brazilian biomes (Amazonia, Atlantic Forest, Caatinga, and Cerrado), as well as from living collections maintained at the Rio de Janeiro Botanical Garden (JBRJ) and the Missouri Botanical Garden (MO). Leaf tissue was preserved in silica gel. All accessions were vouchered, and voucher specimens are deposited at RB and MO herbaria. Voucher information (collection numbers, herbarium codes, and geographic coordinates) is provided in Table S1.

Total genomic DNA was extracted from silica–dried leaf tissue using the DNeasy Plant Mini Kit (Qiagen, Germantown, MD, USA). To isolate enough DNA for sequencing, multiple extractions were performed for some samples, pooled, and concentrated by vacuum centrifugation. An additional purification step was performed using the QIAquick PCR Purification Kit (Qiagen LLC, Germantown, MD, USA). Total DNA was quantified using the Qubit BR Assay Kit.

### 2.2. Library Preparation, Target Enrichment and Sequencing

Genomic libraries were constructed in our laboratory using the NEBNext Ultra II FS DNA Library Prep Kit and Multiplex oligos for Illumina (New England BioLabs Inc., Ipswich, MA, USA). Library concentration and expected size were confirmed using Agilent 2100 BioAnalyzer (Agilent Technologies Inc., Santa Clara, CA, USA), and Qubit dsDNA HS Assay Kit was run on a Qubit 2.0 Fluorometer (Invitrogen, Carlsbad, CA, USA).

Target enrichment was performed via in-solution hybridization capture using the universal Angiosperms353 probe kit [23]. The pooled libraries were submitted to Daicel Arbor Biosciences (Ann Arbor, MI, USA), where hybridization capture was conducted following the manufacturer’s protocols. The enriched libraries were then sequenced on an Illumina NovaSeq 6000 S4 System, generating 150 bp paired-end reads.

### 2.3. Capture Sequencing Data Processing

Raw sequencing reads underwent quality trimming with Trimmomatic v0.39 [32] using parameters “LEADING:3; TRAILING:3; SLIDINGWINDOW:4:20; MINLEN:40”. To serve as a reference for assembly, single-copy orthologous genes from the Angiosperms353 probe set were retrieved for five *Anthurium* species (*A. amnicola* Dressler, *A. formosum* Schott, *A. gracile* (Rudge) Schott, *A. recavum* Croat, and *A. scandens* (Aubl. Engl.) from the Kew Tree of Life Explorer repository [33] using Easy353 v2.0.2 [34].

Sequence capture data processing followed the hybpiper-nf pipeline v1.0.4, with targeted assembly against the Angiosperms353 gene set [22] using the published translated target file (Angiosperms353.FAA). Trimmed reads were binned to genes using BLASTX v2.17.0+ [35] against the translated target file, followed by de novo assembly with SPAdes 3.13.1 [36]. Coding sequences were extracted and flanking non-coding regions were removed using exonerate 2.2 [37].

To ensure data quality, we applied a multi-stage filtering approach. First, samples with exceptionally low mapping ratios (<0.2) were excluded, resulting in the removal of *Anthurium lucidum* and *A. plowmanii*. Second, from the initial 336 targeted genes, we excluded loci with mapping rates below 0.5 in ≥50% of samples. Third, putative paralogous sequences identified by the HybPiper paralog detection pipeline [38] as loci with multiple long contigs (>85% of reference length) were removed to ensure orthology. Because these filters were applied concurrently, the exact number of loci excluded specifically due to paralogy cannot be quantified separately; however, all flagged paralogs were removed. This filtering resulted in a final matrix of 94 loci (27.98% of the original set), with a core set of 68 high-quality loci (20.24%) recovered in ≥90% of samples. To assess the impact of filtering stringency on phylogenetic inference, we compared topologies obtained from the 94-locus and 68-locus datasets; both recovered congruent relationships with comparable support values, indicating that our conclusions are robust to filtering thresholds.

For the concatenation approach, amino acid sequences of HybPiper-reconstructed Angiosperms353 loci were aligned using MAFFT v7.505 [39]. Automated trimming methods (trimAL, ClipKit, CIAlign) were initially tested but produced discordant results and over-trimming artifacts. Therefore, alignments were inspected and trimmed manually using Jalview v2.11.4.0 [40] to remove poorly aligned regions while retaining informative characters, with edited alignments filtered to contain at least eight taxa and 50 amino acids. Summary statistics were calculated using HybPiper scripts (get_seq_lengths.py, hybpiper_stats.py).

### 2.4. Phylogenetic Analysis

Phylogenetic reconstruction was conducted using two complementary approaches widely employed in phylogenomic studies: concatenation (supermatrix) and multispecies coalescent [41]. The concatenation approach assumes a single underlying gene tree and analyzes all loci together, while coalescent-based methods account for gene tree heterogeneity due to processes such as incomplete lineage sorting [41].

After stringent filtering, a final dataset of 68 nuclear loci was used for phylogenetic reconstruction. First, individual gene trees were reconstructed for each locus. The best-fit substitution model for each reconstructed Angiosperms353 locus was independently optimized using ModelTest-NG v0.1.7 [42]. Maximum likelihood (ML) inference was then performed using the ParGenes v1.2.0 pipeline [43], which employs RAxML-NG v1.2.2 [44] for tree inference under the respective best-fit models. Node support was evaluated using 1000 bootstrap replicates

### 2.5. Species Trees

For the concatenation approach, multiple sequence alignments for each locus were concatenated into a supermatrix using SEGUL v0.22.1 [45]. To account for locus-specific heterogeneous evolutionary rates, we performed a partitioned maximum likelihood analysis in RAxML-NG v1.2.2 [44], applying the best-fit substitution models previously inferred for each locus (see Section 2.4). Node support was evaluated with 1000 bootstrap replicates.

For the coalescent-based approach, we used ASTRAL-IV v1.22.4.6 [46]. Input consisted of individual gene trees reconstructed as described in Section 2.4, which were filtered by collapsing branches with bootstrap support below 30% [46]. This threshold follows developer recommendations to reduce gene tree discordance and improve species tree accuracy. To evaluate conflict among individual gene trees and the inferred species tree, we examined the distribution of quartet scores computed by ASTRAL-III, which represent the proportion of gene trees supporting each species tree relationship. Gene tree discordance was further assessed by comparing topologies obtained from concatenated (RAxML-NG) and coalescent-based (ASTRAL-IV) approaches.

The resulting species trees from both analyses were visualized and annotated using the ggtree R package v3.16.0 [47] and further edited using ChiPlot online [48].

### 2.6. Divergence Time Estimation

To estimate divergence times within *Anthurium*, we generated a time-calibrated ultrametric tree from the maximum-likelihood species tree inferred using the supermatrix approach. The ML topology was fixed during divergence dating to focus exclusively on temporal inference while avoiding additional topological uncertainty unrelated to the dating objectives. Divergence dating was performed under the fossilized birth–death (FBD) model in BEAST v2.7.7 [49], using two fossil-based calibration points. Root calibration—The split between subfamily Pothoideae (including the outgroup *Pothos scandens*) and *Anthurium* was calibrated with a normally distributed prior (mean = 112 Ma, SD = 5 Ma), based on early fossil evidence attributed to Pothoideae [50,51]. Crown *Anthurium* calibration—The minimum age of the *Anthurium* crown group was constrained using the fossil †*Petrocardium cerrejonense* (dated to 60–58 Ma) [51]. A lognormal prior (offset = 58 Ma, mean = 1.0, SD = 0.5) was applied to reflect this hard minimum bound while accommodating older plausible divergence ages.

These fossils were selected because they represent the most reliable calibration points currently available for Araceae. The fossil †*Petrocardium cerrejonense* exhibits strong morphological affinity with extant *Anthurium* [51], while the early Pothoideae fossils provide a robust root constraint [50,51]; both have been previously employed as temporal anchors in evolutionary studies of the family.

The analysis employed an uncorrelated relaxed clock model [52] with a birth–death prior on speciation rates. Two independent MCMC runs of 100 million generations were conducted, sampling every 10,000 generations. Convergence and adequate effective sample sizes (ESS > 200) were verified with Tracer v1.7.2 [53]. The maximum clade credibility (MCC) tree was summarized from the combined posterior tree distribution using TreeAnnotator v2.7.7 [52] after discarding 10% of samples as burn-in.

## 3. Results

### 3.1. Gene Recovery

Sequencing yielded 1.25 billion reads (34.56 GB), averaging 7.5 million reads (~1.0 GB) per sample, with per-species totals ranging from 200 MB to 1 GB. The raw dataset initially consisted of 38 samples and 336 targeted genes. Two samples, *Anthurium lucidum* Kunth and *A. plowmanii* Croat, were discarded due to insufficient sequence quality (mapping ratio < 0.2), resulting in a final dataset of 36 samples (Table 1).

**Figure 1:**
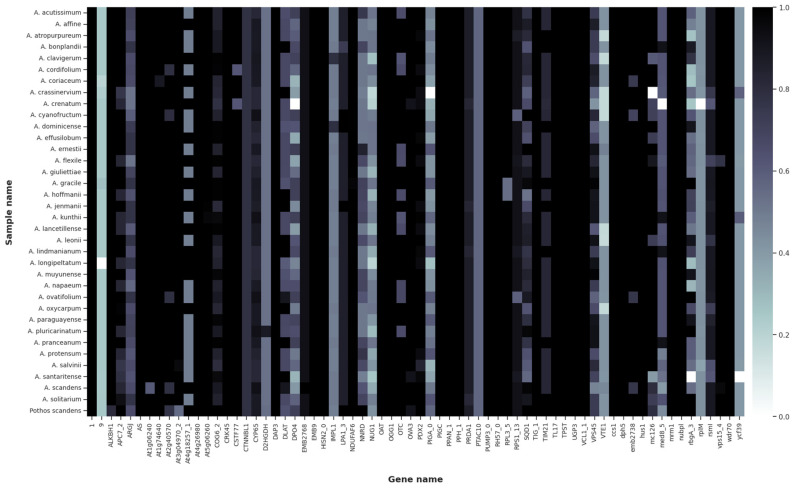
Heatmap showing recovery of 68 nuclear loci (Angiosperms353) across 36 Araceae samples. Each row represents a sample and each column a gene locus. The color coding on the right represents the percentage length recovery of each reference gene relative to the reconstructed *Anthurium* species. In this figure, we filtered the data to display only high-quality mapped genes, with at least 90% of length recovered, totaling 68 genes (20.24%).

Gene recovery was heterogeneous across taxa. While most Amazonian and Atlantic Forest species showed high recovery across loci, some taxa exhibited consistently lower recovery rates, including *A. paraguayense* Croat, *A. ernestii* Engl., and *A. salvinii* Hemsl. (Figure 1). These species showed mapping rates below 0.6 for several loci.

The low overall recovery rate (27.98% of targeted genes) reflects evolutionary divergence between the Angiosperms353 probes, designed primarily from asterids and rosids, and *Anthurium* (monocots). However, the congruence between analyses using different filtering thresholds (94 vs. 68 loci) and the consistency between concatenated and coalescent approaches (see Section 2.4) indicate that the recovered loci provide sufficient phylogenetic signal to resolve relationships within sect. *Pachyneurium*.

### 3.2. Phylogenomic Analysis

**Figure 2:**
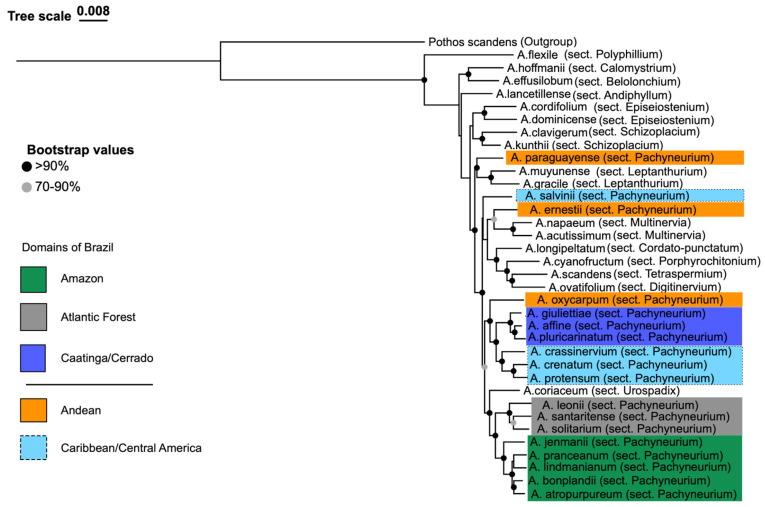
Maximum-likelihood phylogeny inferred from the concatenated 68-locus Angiosperms353 dataset. The analysis includes 35 species of *Anthurium* Schott, with *Pothos scandens* as outgroup. Major clades and sectional affiliations are indicated. Brazilian lineages are color-coded as follows: Amazonian (green), Caatinga/Cerrado (royal blue), and Atlantic Forest (gray). Additional geographic groups are indicated as Caribbean/Central America (light blue) and Andean (orange). Node support values are shown as colored circles (grey = 70–90% bootstrap support; black >90%). Color coding follows Color Universal Design (CUD) principles.

**Figure 3:**
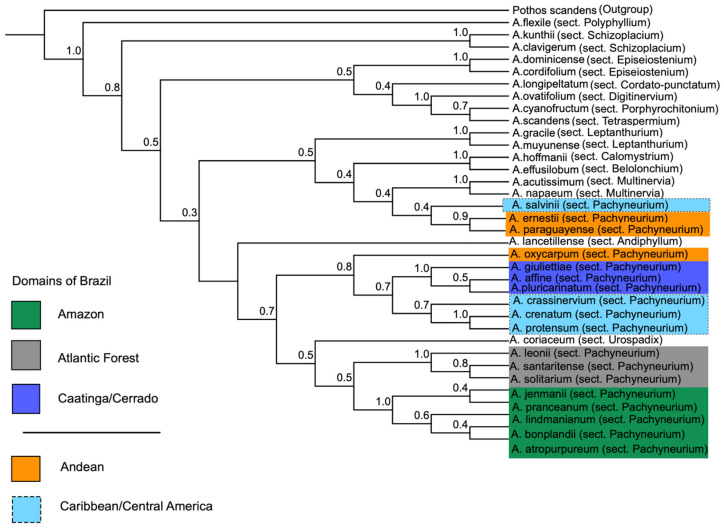
Species tree inferred with ASTRAL-IV under the multi-species coalescent model, based on gene trees from 68 Angiosperms353 loci. The tree includes 35 species of *Anthurium* Schott, with *Pothos scandens* as outgroup. Major clades are labeled. Brazilian lineages are color-coded as Amazonian (green), Caatinga/Cerrado (royal blue), and Atlantic Forest (gray). Caribbean/Central American and Andean lineages are indicated in light blue and orange, respectively. Numbers at nodes represent local posterior probabilities (LPP). Color coding follows Color Universal Design (CUD) principles.

The phylogenomic reconstruction recovered a largely well-resolved phylogeny for 35 *Anthurium* species, including 18 from sect. *Pachyneurium* (Schott) Engl., with *Pothos scandens* (subfamily Pothoideae) as the outgroup. Both concatenated (RAxML-NG; Figure 2) and coalescent-based (ASTRAL-IV; Figure 3) analyses yielded largely congruent topologies. Most nodes received strong statistical support across most major lineages, with bootstrap support (BS) ≥90% in the concatenated analysis and local posterior probabilities (LPPs) ≥0.95 in the coalescent analysis (Table 2). Minor topological incongruences were restricted to a few nodes with moderate or low support and did not affect the primary conclusions regarding major lineages or biogeographic patterns.

Relationships recovered with high statistical support across both analyses (BS ≥ 90%, LPP ≥ 0.95) include (Figure 2 and Figure 3) sect. *Polyphyllium* Engl. *(A. flexile* Schott), inferred as the earliest-diverging lineage; sect. *Episeiostenium* Schott (Engl.) (*A. dominicense* Schott and *A. cordifolium* (Raf.) Kunth); sect. *Schizoplacium* Schott (Engl.) (*A. kunthii* Poepp. and *A. clavigerum* Poepp.); sect. *Multinervia* Croat (Carlsen & Croat) (*A. acutissimum* Engl. and *A. napaeum* Engl.); sect. *Leptanthurium* Schott (Engl.) (*A. gracile* (Rudge) Lindl. and *A. muyunense* Croat), each recovered as distinct and well-supported lineages. Sect. *Tetraspermium* Schott (*A. scandens* (Aubl.) Engl.) was also recovered as a distinct lineage, although relationships among some early-diverging sections received only moderate support.

Within sect. *Pachyneurium*, three Brazilian lineages were consistently recovered with strong support (BS ≥ 90%, LPP ≥ 0.95): the Amazonian lineage (green), the Atlantic Forest lineage (gray), and the Caatinga/Cerrado lineage (royal blue). The Atlantic Forest lineage, comprising *A. leonii* E.G. *Gonç.*, *A. santaritense* Nadruz, and related species, was recovered as sister to *A. coriaceum* (Graham) G.Don (sect. *Urospadix* Schott (Engl.)). The Caatinga/Cerrado lineage includes *A. affine* Schott, *A. pluricarinatum* Camelo, Nadruz, Temponi & Baumgratz, and “*A. giuliettiae*” sp. nov. ined., with the latter consistently resolved as sister to *A. affine* + *A. pluricarinatum* in both analyses. The Caribbean/Central American lineage, including the type species of sect. *Pachyneurium*, *A. crenatum* (L.) Kunth, together with *A. protensum* Schott and *A. crassinervium* (Jacq.) Schott, was also strongly supported.

Moderate support (BS 70–90%, LPP 0.80–0.90) was observed for relationships among some deeper nodes, including the placement of sect. *Tetraspermium* and the relative order of early-diverging sections (Figure 2 and Figure 3).

Instability between concatenated and coalescent analyses, coupled with low support values (BS < 70%, LPP < 0.80), was detected for the following taxa (Figure 2 and Figure 3): *Anthurium paraguayense* Croat placed within the Andean lineage in the concatenated analysis as sister to the clade including Caribbean/Central American species in the coalescent tree; *Anthurium ernestii* Engl. showing conflicting placements among early-diverging lineages; and *Anthurium salvinii* Hemsl. grouped with Caribbean/Central America taxa in the concatenated analysis in the coalescent tree (LPP = 0.40).

Relationships among *A. oxycarpum* Poepp. and other Andean taxa also exhibited low support and topological variation. These unstable placements may reflect the lower gene recovery rates observed for these taxa (see Section 3.1; Figure 1), which may have limited phylogenetic signal.

### 3.3. Time-Calibrated Tree

**Figure 4:**
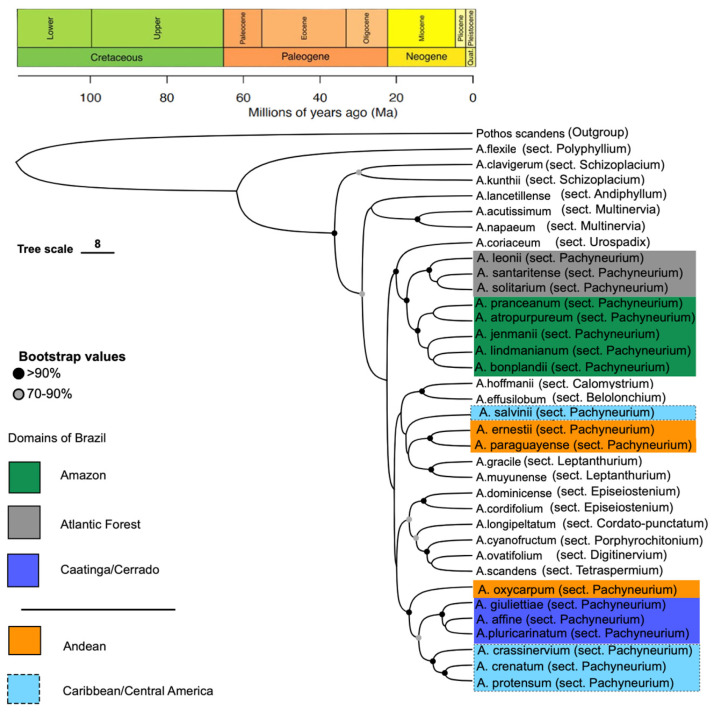
Time-calibrated ultrametric phylogeny of *Anthurium* derived from the maximum-likelihood supermatrix analysis. The topology was fixed during divergence time estimation. Major clades are labeled. Brazilian lineages are color-coded as Amazonian (green), Caatinga/Cerrado (royal blue), and Atlantic Forest (gray). Caribbean/Central American and Andean lineages are shown in light blue and orange, respectively. Divergence times were estimated using two fossil calibrations (see Methods). Horizontal bars represent 95% highest posterior density (HPD) intervals. Node circles indicate bootstrap support from the ML analysis (grey = 70–90%; black > 90%). Color coding follows Color Universal Design (CUD) principles.

The time-calibrated ultrametric phylogeny (Figure 4) is topologically congruent with the major, strongly supported relationships recovered in both the concatenated maximum likelihood tree (BS ≥ 90%; Figure 2) and the coalescent-based species tree (LPP ≥ 0.95; Figure 3). Nodes with moderate support (bootstrap values between 70 and 90%) in the ML analysis appear collapsed in the chronogram.

Additionally, relationships involving taxa with low support in the coalescent analysis, such as sect. *Cordato-punctatum* Croat & Carlsen (*A. longipeltatum* Matuda; LPP = 0.40), are not resolved in the time-calibrated tree. Consequently, while being robust for the major lineages, relationships among lineages or taxa with lower statistical support remain unresolved.

### 3.4. Origins and Initial Divergences of Genus Anthurium

The crown age of *Anthurium* was estimated at approximately 62 Ma (95% HPD: 58–65 Ma), corresponding to the Paleocene (Figure 4). The crown age of subfamily Pothoideae was estimated at approximately 112 Ma (95% HPD: 105–120 Ma).

### 3.5. Origin and Initial Divergences of the Section Pachyneurium and Its Brazilian Lineages

The main divergence events among the sampled lineages of sect. *Pachyneurium* occurred during the Miocene (c. 20–10 Ma; Figure 4). Divergence within the three principal Brazilian lineages is estimated as follows. Amazonian lineage: divergence dates to the Middle Miocene (c. 15–12 Ma).

Atlantic Forest lineage: divergence dates to the Middle–Late Miocene (c. 12–8 Ma). Caatinga/Cerrado lineage: divergence dates from the Late Miocene to the Pliocene (c. 8–3 Ma). Divergence within the Caribbean/Central American lineage and the Andean clade also dates to the Miocene. Credibility intervals for these nodes broadly overlap within this period (Figure 4).

## 4. Discussion

### 4.1. Implications for the Systematics and Taxonomy of Section Pachyneurium

The non-monophyly of sect. *Pachyneurium* recovered in our analyses has implications for the infrageneric classification of *Anthurium*. This finding is consistent with earlier molecular studies that suggested the section may not represent a natural group [7,8], and provides new evidence from previously unsampled Brazilian taxa.

The distribution of species formerly placed in *Pachyneurium* across multiple distinct lineages suggests that the morphological characters traditionally used to diagnose the section (rosulate habit, involute prefoliation, primary lateral veins free and collective vein absent) are homoplastic. Such morphological convergence may be explained by adaptive responses to similar environmental pressures, particularly in seasonally dry habitats, and is well-documented in other megadiverse plant genera [54]. Our findings suggest that *Anthurium* represents another case in which vegetative morphology may be misleading when used to recognize phylogenetic clades.

The sister relationship between an Atlantic Forest lineage traditionally assigned to *Pachyneurium* and *A. coriaceum* (sect. *Urospadix*) is particularly noteworthy. Despite marked morphological differences, their close evolutionary affinity indicates that historical biogeography may be as important as morphology in shaping relationships within the genus. This finding highlights the value of phylogenomic data in revealing evolutionary relationships that are not apparent from morphology alone.

Given that our sampling does not encompass the full diversity of sect. *Pachyneurium*, these results are not intended as a formal taxonomic revision. Rather, they provide an evolutionary framework to guide future studies with expanded sampling and detailed morphological reassessment.

### 4.2. Biogeographic Insights and Diversification of Brazilian Lineages of Section Pachyneurium

The temporal and geographic patterns recovered in our phylogeny invite comparison with the known geoclimatic history of South America. However, given that our sampling includes only 15% of the section’s total diversity (18 of ~120 species) and lacks comprehensive representation of Caribbean, Central American, and Andean lineages, the following interpretations are presented as testable hypotheses to guide future studies with expanded taxon sampling.

The diversification of the three main Brazilian lineages within sect. *Pachyneurium* occurred during the Miocene and Pliocene (20–3 Ma), a period of significant environmental change in South America. The sister relationship between Atlantic Forest and Amazonian lineages is consistent with a vicariance hypothesis, in which a previously continuous distribution became fragmented. The Miocene expansion of drier vegetation across the South American dry diagonal [55,56,57,58,59] has been invoked to explain similar patterns in other plant groups and may represent a plausible mechanism for the separation of these two lineages.

Diversification within the Amazonian lineage coincides temporally with the consolidation of the modern Amazon Basin and its associated humid forests [60,61,62]. This period of landscape stabilization may have created ecological opportunities for in situ radiation. The Miocene Amazonian wetland system (Pebas System, ~20–10 Ma) has been hypothesized to have acted as a biogeographic barrier for terrestrial organisms [63], potentially promoting allopatric diversification, though testing this hypothesis would require denser sampling across the Amazon basin.

The Caatinga/Cerrado lineage represents a more recent diversification, coinciding with Neogene aridification in eastern South America. The current disjunct distributions of species in this lineage some restricted to humid forest enclaves (*brejos de altitude*), others to coastal *restingas* [12,14]—are compatible with Pleistocene refugia models [62], suggesting that climatic oscillations may have shaped their genetic structure. However, population-level data would be necessary to test this hypothesis rigorously.

Beyond Brazil, the Miocene diversification of Caribbean/Central American and Andean lineages raises the hypothesis that the *Pachyneurium* may have arisen independently in multiple lineages across the Neotropics in response to similar environmental conditions [8,9,55]. This pattern could be consistent with the geological history of western Amazonia, where intermittent connections between the Andes, the Caribbean, and the Amazon Basin during the Miocene may have facilitated episodic biotic exchange [63].

Formal biogeographic reconstruction using model-based approaches (e.g., DEC or DIVA) was not performed here due to incomplete sampling across the full geographic range of the section. Therefore, our current biogeographic hypotheses are presented as propositions to be tested with expanded datasets that include broader representation of Caribbean, Central American, and Andean lineages, as well as population-level sampling within Brazil.

### 4.3. Methodological Insights, Limitations and Conservation Implications

The main methodological limitation was the lower-than-expected recovery of nuclear loci using the Angiosperms353 probe set (27% of targeted genes). This likely reflects divergence between the asterid/rosid-derived probes [23] and *Anthurium* (monocots). Future studies may benefit from lineage-specific probe design to improve capture efficiency.

Despite this limitation, the recovered loci resolved relationships within sect. *Pachyneurium* with strong support for most nodes, demonstrating that partial datasets from universal probe sets can provide meaningful phylogenetic signal in rapidly radiating lineages.

Our sampling included 11 Brazilian species (eight endemics), representing 52% of Brazilian taxa and 15% of the section’s total diversity. While this design meets our objective of testing sectional monophyly, the use of a single accession per species does not capture intraspecific variation or cryptic diversity, which would require population-level sampling. Divergence time estimates based on only two fossil calibrations [50,51] represent a temporal hypothesis to be tested with additional fossil evidence in future studies. The potential effects of incomplete lineage sorting and hybridization on gene tree discordance remain unresolved; species network analyses would be necessary to distinguish between deep coalescence and introgression.

Despite these limitations, this study contributes to reducing two fundamental shortfalls in biodiversity knowledge [64]. First, we address the Darwinian shortfall by providing a resolved phylogenomic hypothesis for sect. *Pachyneurium*, contributing to the Plant Tree of Life (PAFTOL) initiative. Second, by integrating phylogeny with divergence times and distribution data, we begin to address the Wallacean shortfall, offering testable hypotheses for vicariance and dispersal in *Anthurium*.

Finally, our framework informs conservation by identifying evolutionarily distinct lineages. Within the Northeastern Lineage, the widespread *A. affine* contrasts with its narrowly endemic sister species *A. pluricarinatum* and *A. giuliettiae* sp. nov. ined., which are restricted to threatened habitats (*brejos de altitude* and *restingas*). The Atlantic Forest lineage also includes the endangered *A. santaritense*. These observations highlight the need for population-level studies to assess genetic diversity within these narrowly endemic species.

## 5. Conclusions

This study provides the first phylogenomic hypothesis for Brazilian species of *Anthurium* sect. *Pachyneurium*. Our results demonstrate that the section is not monophyletic as traditionally circumscribed; instead, the Brazilian species form three geographically structured lineages: Amazonian, Atlantic Forest, and Caatinga/Cerrado (Northeastern). The Atlantic Forest lineage is sister to *A. coriaceum* (sect. *Urospadix*), revealing an unexpected evolutionary relationship. Divergence of these lineages occurred during the Miocene, coinciding with major geoclimatic events.

Beyond these findings, this study offers methodological and taxonomic contributions. It shows that partial locus recovery from Angiosperms353 can resolve relationships in rapidly radiating lineages, providing a reference for similar groups. Taxonomically, it identifies the rosulate habit and involute prefoliation as homoplastic, laying groundwork for future revision of Brazilian *Pachyneurium* and broader sectional recircumscription.

This work is part of a larger project that will expand sampling to approximately 400 *Anthurium* species and develop a genus-specific bait kit. Future steps include applying this toolkit across the Caribbean, Central America, and the Andes, combined with population-level data to address incomplete lineage sorting and hybridization.

By advancing knowledge of a megadiverse genus, this study contributes to the Plant Tree of Life initiative and highlights the value of integrating phylogenomics with taxonomy and conservation.

## Figures and Tables

**Table 1:** Summary of sequence capture filtering statistics.

Description	Value
**Original number of samples**	38
**Original number of genes**	336
**Samples rejected (mapping ratio < 0.2)**	2 (*A. lucidum*, *A. plowmanii*)
**Genes rejected (mapping rate < 0.5 in ≥ 50% samples)**	242 (72.02%)
**Genes retained (mapping rate ≥ 0.5)**	94 (27.98%)
**Final number of samples**	36
**Final number of genes**	94
**High-quality genes (mapping rate >0.9)**	68 (20.24%)

**Table 2:** Summary of well-supported lineages recovered within *Anthurium* sect. *Pachyneurium*, including representative species and statistical support from maximum likelihood (ML) bootstrap (BS) and ASTRAL local posterior probabilities (LPPs). Taxa showing unstable placement across analyses are indicated separately. High support: BS ≥ 90% and LPP ≥ 0.95; low support/unstable: BS < 70% and LPP < 0.80.

Lineage	Representative Species	ML Support (BS)	ASTRAL Support (LPP)
Amazon	*A. atropurpureum*, *A. bonplandii*, *A. jenmanii*, *A. lindmanianum*, *A. pranceanum*	≥90%	≥0.95
Atlantic Forest	*A. leonii*, *A. santaritense*, *A. solitarium*	≥90%	≥0.95
Caatinga/Cerrado	*A. affine*, *A. giuliettiae* sp. nov. ined., *A. pluricarinatum*	≥90%	≥0.95
Caribbean/Central America	*A. crassinervium*, *A. crenatum*, *A. protensum*	≥90%	≥0.95
Unstable taxa	*A. ernestii*, *A. oxycarpum*, *A. paraguayense*, *A. salvinii*	<70%	<0.80

## Data Availability

The original contributions presented in this study are included in the article/Supplementary Materials. The Angiosperms353 reference probe sequences used for target enrichment are publicly available on GitHub at: https://github.com/mossmatters/Angiosperms353 accessed on 3 March 2025. The raw sequence reads have been deposited in the NCBI Sequence Read Archive (SRA) under the BioProject accession number PRJNA1413566 (https://www.ncbi.nlm.nih.gov/sra/PRJNA1413566, accessed on 3 March 2025). Additionally, the voucher information table (Table S1) and other supporting data are openly available in Figshare at https://doi.org/10.6084/m9.figshare.31456999.

## Supplementary Materials

The following supporting information can be downloaded at Figshare: https://doi.org/10.6084/m9.figshare.31456999. Table S1: List of taxa, section, voucher information, herbarium and country used in the phylogenomic analyses in this study.
